# Extracellular CIRP Upregulates Proinflammatory Cytokine Expression via the NF-kappaB and ERK1/2 Signaling Pathways in Psoriatic Keratinocytes

**DOI:** 10.1155/2022/5978271

**Published:** 2022-09-06

**Authors:** Xiu Zhang, Shengbang Wang, Wei Wang, Liumei Song, Shuo Feng, Jingping Wang, Tong Kang, Peiwen Yang, Ning Wang, Pengju Yang, Ruimin Bai, Yongping Shao, Yan Zheng

**Affiliations:** ^1^Department of Dermatology, The Second Affiliated Hospital of Xi'an Jiaotong University, Xi'an 710004, China; ^2^Department of Dermatology, The First Affiliated Hospital of Xi'an Jiaotong University, Xi'an 710061, China; ^3^Frontier Institute of Science and Technology, Xi'an Jiaotong University, Xi'an 710049, China

## Abstract

Psoriasis is a chronic inflammatory skin disease, and elevation of proinflammatory cytokine levels is a critical driver of the pathogenesis of psoriasis. Extracellular cold-inducible RNA-binding protein (eCIRP) has been shown to play a role in various acute and chronic inflammatory diseases. C23, a short peptide derived from CIRP, competitively binds CIRP receptors and reduces damage in inflammatory diseases. However, the effect of eCIRP in psoriasis has not been studied. In the present study, we investigated the role of eCIRP in the expression of proinflammatory cytokines in keratinocytes. Our data show that eCIRP expression was increased in the sera of psoriasis patients and imiquimod- (IMQ-) induced psoriatic mice and cells stimulated with proinflammatory cytokines (IL-1*α*, IL-17A, IL-22, oncostatin M, and TNF-*α*; mix M5). Recombinant human CIRP (rhCIRP) promoted the expression of the proinflammatory cytokines TNF-*α*, IL-6, and IL-8 and the activation of NF-kappaB (NF-*κ*B) and ERK1/2 in cultured keratinocytes. We then found that the above effects of eCIRP could be blocked by C23 in both normal keratinocytes and M5-stimulated psoriatic keratinocytes. In addition, in vivo experiments revealed that C23 could effectively ameliorate IMQ-induced psoriatic dermatitis. TNF-*α* and IL-6 mRNA expressions were reduced in the skin lesions of mice with C23-treated IMQ-induced psoriasis, and this effect was accompanied by inhibition of the NF-*κ*B and ERK1/2 signaling pathways. In summary, eCIRP plays an important role in the pathogenesis of psoriasis and may become a new target for psoriasis treatment.

## 1. Introduction

Psoriasis is a common, chronic, recurrent, inflammatory skin disease characterized by erythema and scaling that occurs in approximately 125 million people worldwide, generating a great disease burden [1–3]. Dysregulated inflammatory pathways and increased levels of proinflammatory cytokines, such as TNF-*α* [3, 4], IL-6 [5, 6], and IL-8 [7, 8], are critical in the pathogenesis of psoriasis. Psoriasis is a currently incurable chronic skin disease that greatly reduces patients' quality of life. The treatment of psoriasis mainly includes topical therapies such as topical corticosteroids; vitamin D analogs; keratolytic agents; phototherapy; systemic therapies such as biologics targeting the proinflammatory cytokines TNF-*α*, IL-17, and IL-23; and oral systemic therapy [3]. Multiple treatments for psoriasis are available, but there is still no cure. Therefore, it remains important to further study the pathogenesis of psoriasis and to find new therapeutic targets to obtain clinical treatments for psoriasis with better efficacy and greater safety.

Cold-inducible RNA-binding protein (CIRP) is a member of the glycine-rich RNA-binding protein (GRP) family, first identified by Nishiyama et al. [9] during the study of gene transcription. CIRP undergoes cytoplasm/nucleus redistribution and extracellular secretion in response to different stresses [10]. Extracellular CIRP (eCIRP) acts as a danger-associated molecular pattern (DAMP) to promote inflammatory responses that play an important role in acute and chronic inflammation, such as sepsis [11, 12], rheumatoid arthritis (RA) [13], and osteoarthritis (OA) [14]. Recently, researchers have identified C23 as a potential CIRP antagonist that can inhibit eCIRP-induced phagocyte secretion of TNF-*α* [10]. Although eCIRP has shown proinflammatory effects in many inflammatory diseases, its effect on psoriasis has not been studied.

Here, eCIRP expression was increased in the sera of psoriasis patients and imiquimod- (IMQ-) induced psoriatic mice, and it was also elevated in the media of psoriatic keratinocytes. Upon coincubation of recombinant human CIRP (rhCIRP) and/or C23 with keratinocytes, we found that rhCIRP could upregulate proinflammatory cytokine expression and activate the NF-kappaB (NF-*κ*B) and ERK1/2 signaling pathways in keratinocytes, while C23 was able to inhibit the above effects of rhCIRP. Our results showed that the eCIRP level was positively correlated with psoriasis and that eCIRP may be involved in the pathogenesis of psoriasis by upregulating proinflammatory cytokine expression via activation of the NF-*κ*B and ERK1/2 signaling pathways.

## 2. Materials and Methods

### 2.1. Human Serum Sample Collection

Serum samples were obtained from healthy volunteers (6 males and 4 females) and patients with psoriasis (22 males and 8 females) from the Department of Dermatology, The Second Affiliated Hospital of Xi'an Jiaotong University who had received no medical treatment within the prior 4 weeks. The study followed the Declaration of Helsinki, and the study protocol was approved by the Institutional Ethics Committee of Xi'an Jiaotong University. All recruited participants in this study provided written informed consent.

### 2.2. IMQ-Induced Psoriatic Mouse Model

Male BALB/c mice (6-8 weeks; 18-20 g), free of specific pathogens, were used in the experiments. The animals were obtained from the Animal Center of Xi'an Jiaotong University and were kept under standard laboratory conditions under a 12-hour light to dark cycle with an ambient temperature of 24-26°C. All animal experiments were approved by the Institutional Animal Care and Use Committee of Xi'an Jiaotong University. The mice received a daily topical dose of 62.5 mg of commercially available IMQ cream (5%; Sichuan Mingxin Pharmaceutical Co., Ltd.) on the shaved back for 7 consecutive days, as described previously [15, 16]. In the therapeutic group, the mice were treated with calcipotriol (C, 40 mg/cm^2^, Leo Pharmaceutical Co., Ltd.) daily after IMQ application. In the control group, an equal amount of Vaseline (Nanchang Baiyun Pharmaceutical Co., Ltd.) was applied. After the experiment, all mice were euthanized, and their serum and skin samples were stored for determination and further experiments.

### 2.3. Cell Culture

HaCaT and HEKa cells (human keratinocyte cell lines) were cultured in DMEM containing 10% fetal bovine serum and 1% penicillin/streptomycin at 37°C in a humidified atmosphere with 5% CO_2_. Cells were stimulated with 10 ng/ml M5 (IL-1*α*, IL-17A, IL-22, oncostatin M, and TNF-*α*; PeproTech, USA) for 24 hours at 37°C to mimic a psoriasis-like environment in keratinocytes [17–20]. Cells were then either left untreated or pretreated with 1 *μ*g/ml C23 (GRGFSRGGGDRGYGG synthesized from GenScript, Piscataway, NJ; dissolved in phosphate-buffered saline) for 30 minutes and then were stimulated with 1 *μ*g/ml rhCIRP (APG886Hu01, Cloud-Clone Corp, Texas, USA) for 1 hour. After rhCIRP stimulation, cells were collected for qRT-PCR and western blot analysis.

### 2.4. Enzyme-Linked Immunosorbent Assay (ELISA)

The concentrations of eCIRP from human serum and culture supernatant of HaCaT and HEKa cells were detected through standard ELISA using ELISA kits according to the manufacturer's instructions (Human CIRP ELISA kit, SEG886Hu, Cloud-Clone Corp, Texas, USA), and the eCIRP concentration in mouse serum was detected through standard ELISA using ELISA kits according to the manufacturer's instructions (Mouse CIRP ELISA kit, JM-12363M2, Jingmei Biotechnology, Jiangsu, China).

### 2.5. RNA Extraction and qRT-PCR

Total RNA was extracted from the samples using TRIzol reagent (15596018, Invitrogen, Carlsbad, CA), and cDNA was generated and then amplified with Evo M-MLV RT Premix for qPCR (AG11706, Accurate Biotechnology, Hunan, China), followed by qRT-PCR on an ABI StepOnePlus Real-Time PCR System (Applied Biosystems, Foster City, CA) using a Premix Pro TaqHS qPCR Kit (Accurate Biotechnology, AG11701, Hunan, China). The primers (see Supplementary Table (available here)) were synthesized by Sangon Biotech (Shanghai, China). Relative quantification was calculated using the 2^–*ΔΔ*CT^ formula, and the expression data were normalized with GAPDH or *β*-actin gene expression.

### 2.6. Western Blot Analysis

Total protein was extracted from cells or skin tissues using RIPA lysis buffer (PL001; ZHONGHUIHECAI, Xi'an, China) supplemented with protease inhibitor (4693159001; Roche, Basel, Switzerland) and phosphatase inhibitor (4906845001; Roche). The protein content was quantified using a BCA Protein Assay Kit (23227; Thermo Fisher Scientific, Waltham, MA). Protein expression levels were analyzed by standard western blot protocols. Protein samples were separated by SDS-PAGE and transferred onto polyvinylidene fluoride membranes (03010040001; Roche, Basel, Switzerland). The membranes were blocked with 5% nonfat milk for 1 hour and incubated with primary antibodies at 4°C overnight and horseradish peroxidase-conjugated secondary antibodies for 1 hour at room temperature. Signals were detected using an ECL Detection Kit (WBKlS0100; Millipore) and developed using a ChemiDoc MP Imaging System (Bio-Rad, Hercules, CA). *β*-Tubulin was used as an internal control. Antibodies against the following proteins were used for western blot analysis: NF-*κ*B p65 (10745-1-AP) and *β*-tubulin (10094-1-AP) from Proteintech (Rosemont, IL) and phospho-NF-*κ*B p65 (Ser536) (93H1), phospho-ERK1/2 (Thr202/Tyr204) (4370), and ERK1/2 (4695) from Cell Signaling Technology (Danvers, MA).

### 2.7. In Vivo Administration of C23

**Figure 1:**
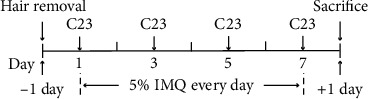
Experimental schedule of the use of IMQ and C23 in mice.

Mice were randomly divided into the control group, IMQ-induced group (IMQ), and C23-treated IMQ group (IMQ+C23). Mice in the IMQ+C23 group received an intraperitoneal injection of 8 mg/kg BW C23 on days 1, 3, 5, and 7 after IMQ application began. The dosage of C23 was determined based on previous adult murine models of renal ischemia–reperfusion (I/R) injury [21] and sepsis [22]. Mice in the IMQ group received an equivalent volume of normal saline. The experimental schedule of using IMQ and C23 in mice is shown in Figure 1.

### 2.8. Histology

Skin tissues were paraffin embedded and sectioned. Hematoxylin and eosin (H&E) staining was performed following a standard staining procedure. H&E-stained sections were scanned by a Hamamatsu digital pathology system, and epidermal thickness was determined using NDP.view software. The distance from the basal layer to the base of the stratum corneum was measured. Six randomly selected areas in each section were measured, and the mean values were calculated.

### 2.9. Statistical Analysis

Data are presented as the mean ± SEM. Statistical analysis was carried out using SPSS Statistics version 26.0 software (SPSS, Chicago, IL, USA). Student's *t* test or the Mann-Whitney *U* test was used for comparisons between two groups, and one-way ANOVA followed by the Student-Newman-Keuls post hoc test was used for comparisons among more than two groups. Differences for which *P* < 0.05 were considered statistically significant.

All methods were performed in accordance with the relevant guidelines and regulations.

## 3. Results

### 3.1. eCIRP Levels Are Elevated in Psoriasis

**Figure 2:**
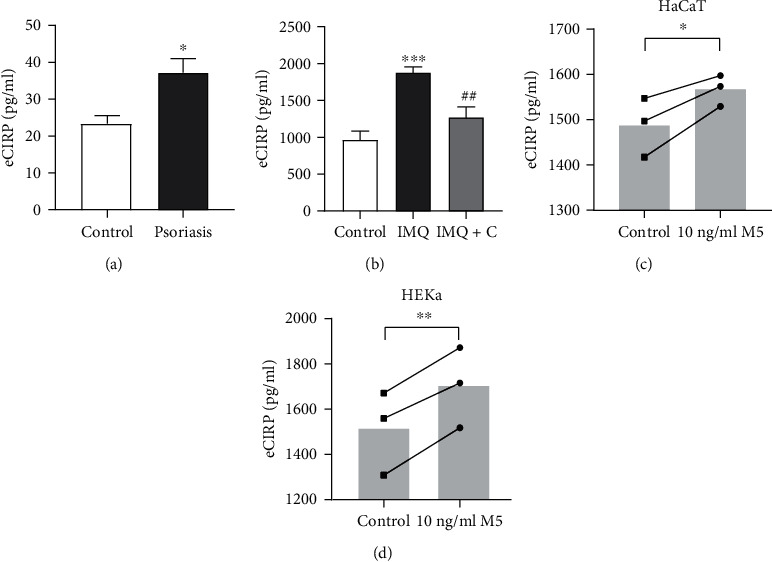
Levels of eCIRP in psoriasis. (a) Detection of eCIRP serum levels in psoriasis patients (*n* = 30) and normal controls (*n* = 10) by ELISA. (b) eCIRP levels in the sera of IMQ-induced psoriatic mice were measured by ELISA (*n* = 5 mice/group). IMQ: IMQ-induced psoriatic mice; IMQ+C: IMQ-induced psoriatic mice treated with calcipotriol. HaCaT (c) and HEKa (d) cells were stimulated with M5 (10 ng/ml each of IL-1*α*, IL-17A, IL-22, oncostatin M, and TNF-*α*) for 24 hours. The eCIRP levels in the culture supernatants were determined using a commercial ELISA kit. Data are expressed as the mean ± SEM. ^∗^*P* < 0.05, ^∗∗^*P* < 0.01, and ^∗∗∗^*P* < 0.001 vs. control; ^##^*P* < 0.01 vs. IMQ.

To explore the level of eCIRP in psoriasis, the serum eCIRP concentrations in psoriasis patients (*n* = 30) and healthy controls (*n* = 10) were measured using a CIRP human serum ELISA kit, and the results showed that serum eCIRP concentrations were elevated in patients with psoriasis compared to the healthy control group (Figure 2(a)). Additionally, serum eCIRP was elevated in IMQ-treated psoriatic mice (*n* = 5) compared to the control mice (*n* = 5) (Figure 2(b)). Calcipotriol, the vitamin D derivative of choice for the treatment of psoriasis, has been shown to reduce erythema, scaling, and epidermal hyperplasia in psoriasis [23]. To clarify the changes in eCIRP levels after treatment, IMQ-induced psoriatic mice were treated topically with calcipotriol for 7 days, and we found that the eCIRP level decreased after calcipotriol treatment (*n* = 5) (Figure 2(b)). Furthermore, to determine whether the release of eCIRP would be changed under the psoriasis-like environment in keratinocytes, HaCaT and HEKa cell lines were stimulated with M5 for 24 hours, which has been demonstrated as an in vitro model of psoriasis. We measured the expression level of eCIRP in the media of M5-stimulated keratinocytes by ELISA, and the results showed that the release of eCIRP in M5-stimulated keratinocytes was greater than that in the control group (Figures 2(c) and 2(d)).

### 3.2. Regulatory Effects of eCIRP on Proinflammatory Cytokine Expression and Signaling Pathway Activation in Keratinocytes

**Figure 3:**
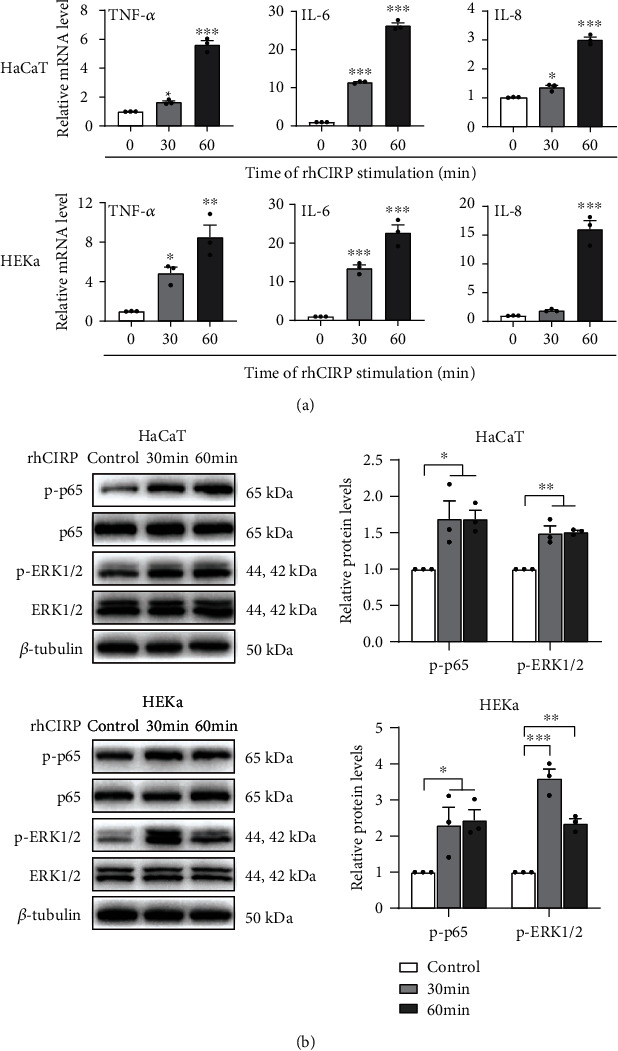
Effects of eCIRP on proinflammatory cytokine expression and the phosphorylation of signaling molecules. (a) rhCIRP caused the time-dependent upregulation of TNF-*α*, IL-6, and IL-8 in HaCaT and HEKa cells at 30 min and 60 min after 1 *μ*g/ml rhCIRP stimulation; the mRNA levels in the two cell lines were measured by qRT-PCR. (b) HaCaT and HEKa cells were treated with 1 *μ*g/ml rhCIRP for 30 and 60 min, and the phosphorylation of NF-*κ*B p65 and ERK1/2 was analyzed by western blotting. Data are expressed as the mean ± SEM. *n* = 3/group. ^∗^*P* < 0.05, ^∗∗^*P* < 0.01, and ^∗∗∗^*P* < 0.001 vs. control.

We stimulated HaCaT and HEKa cells with rhCIRP and found that rhCIRP time-dependently upregulated the expression of the proinflammatory cytokines TNF-*α*, IL-6, and IL-8 in both cell lines, indicating that eCIRP may promote the formation of an inflammatory microenvironment (Figure 3(a)). It has been reported that the NF-*κ*B and ERK1/2 signaling pathways are closely associated with psoriasis development [24, 25]. To elucidate the molecular mechanism underlying the effect of eCIRP on the keratinocyte inflammatory response, we detected the relative protein levels of NF-*κ*B p65 and ERK1/2 by western blotting. As shown in Figure 3(b), stimulation with rhCIRP increased the phosphorylation of NF-*κ*B p65 and ERK1/2 in both HaCaT and HEKa cells. Therefore, eCIRP may be involved in regulating the keratinocyte inflammatory response by the NF-*κ*B p65 and ERK1/2 signaling pathways.

### 3.3. Effects of C23 on Psoriatic Keratinocytes Stimulated with eCIRP

**Figure 4:**
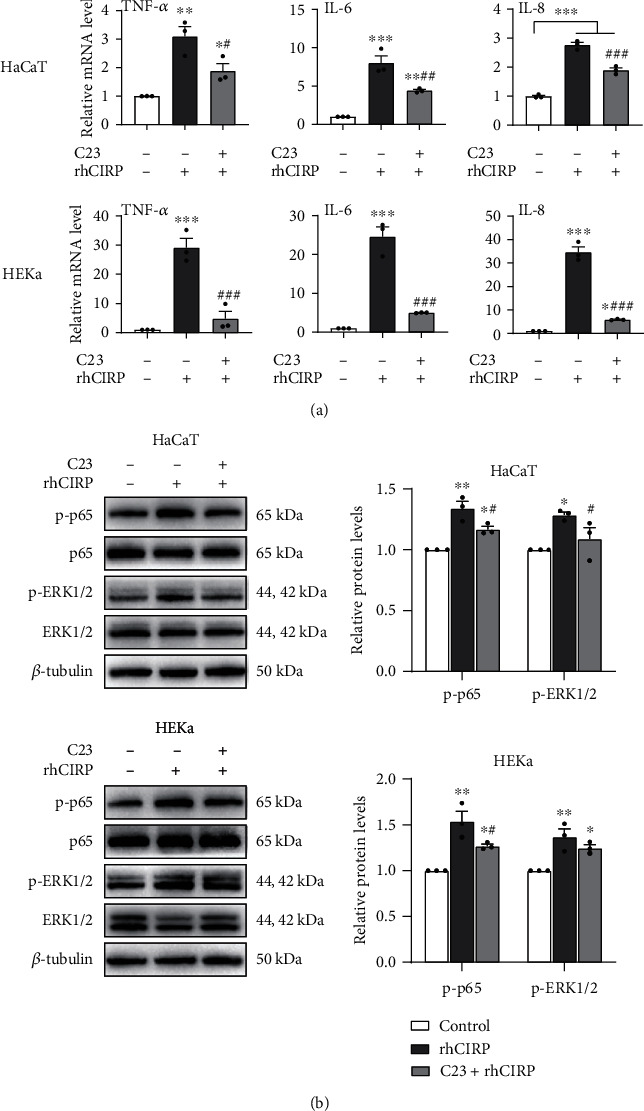
C23 inhibited the eCIRP-induced upregulation of proinflammatory cytokine expression and activation of signaling pathways. (a) C23 inhibited TNF-*α*, IL-6, and IL-8 production in HaCaT and HEKa cells stimulated with rhCIRP. Cells were pretreated with 1 *μ*g/ml C23 for 30 minutes and then were incubated with 1 *μ*g/ml rhCIRP for 1 hour. RNA was extracted for analysis by qRT-PCR. (b) C23 inhibited the phosphorylation of NF-*κ*B p65 and ERK1/2 in HaCaT and HEKa cells stimulated with rhCIRP. Cells were pretreated with 1 *μ*g/ml C23 for 30 minutes and then were incubated with 1 *μ*g/ml rhCIRP for 1 hour. Cells were lysed for western blot analysis of the indicated proteins. The phosphorylation of NF-*κ*B p65 and ERK1/2 was analyzed by western blot analysis. Data are expressed as the mean ± SEM. *n* = 3/group. ^∗^*P* < 0.05, ^∗∗^*P* < 0.01, and ^∗∗∗^*P* < 0.001 vs. control; ^#^*P* < 0.05, ^##^*P* < 0.01, and ^###^*P* < 0.001 vs. rhCIRP alone.

**Figure 5:**
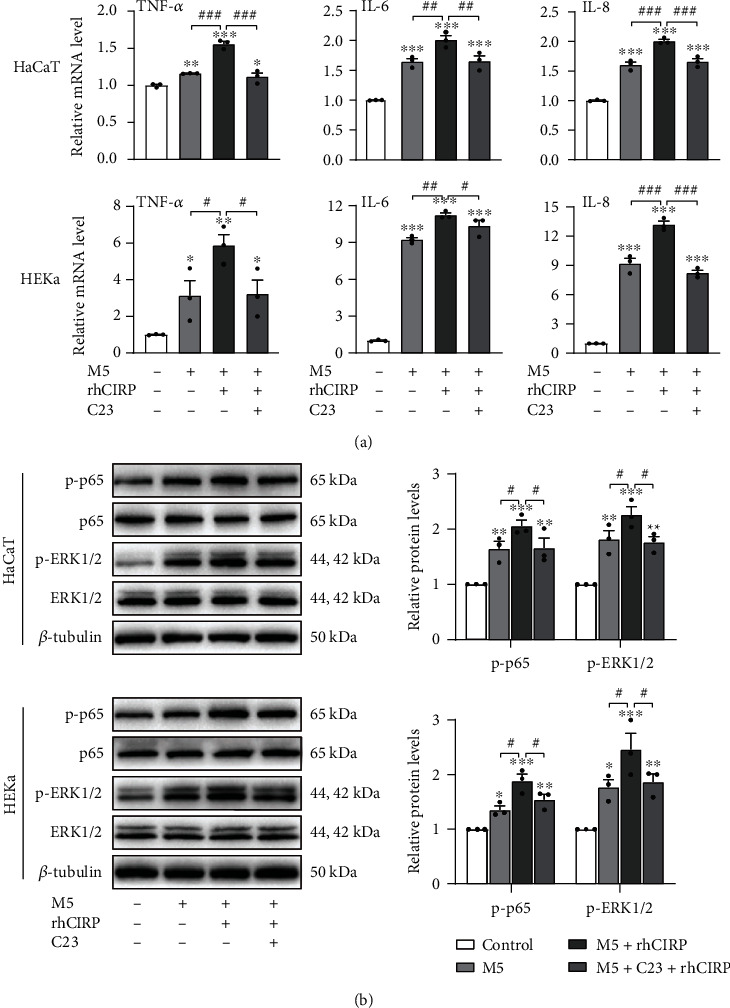
C23 inhibited the eCIRP-induced upregulation of proinflammatory cytokine expression and activation of signaling pathways in M5-stimulated psoriatic keratinocytes. (a) C23 inhibited TNF-*α*, IL-6, and IL-8 production in psoriatic keratinocytes stimulated with rhCIRP. Cells were stimulated with M5 for 24 hours, pretreated with 1 *μ*g/ml C23 for 30 minutes, and then incubated with 1 *μ*g/ml rhCIRP for 1 hour. RNA was extracted for analysis by qRT-PCR. (b) C23 inhibited the phosphorylation of NF-*κ*B p65 and ERK1/2 in psoriatic keratinocytes stimulated with rhCIRP. Cells were stimulated with M5 for 24 hours, pretreated with 1 *μ*g/ml C23 for 30 minutes, and then incubated with 1 *μ*g/ml rhCIRP for 1 hour. Cells were lysed for western blot analysis of the indicated proteins. The phosphorylation of NF-*κ*B p65 and ERK1/2 was analyzed by western blot analysis. Data are expressed as the mean ± SEM. *n* = 3/group. ^∗^*P* < 0.05, ^∗∗^*P* < 0.01, and ^∗∗∗^*P* < 0.001 vs. control; ^#^*P* < 0.05, ^##^*P* < 0.01, and ^###^*P* < 0.001.

As previously shown, keratinocytes exposed to rhCIRP showed significant increases in TNF-*α*, IL-6, and IL-8 production compared to the basal levels. However, pretreatment of keratinocytes with C23 significantly reduced rhCIRP-induced TNF-*α*, IL-6, and IL-8 expressions compared to that in keratinocytes administered rhCIRP only (Figure 4(a)). Likewise, we found that rhCIRP-induced phosphorylation of NF-*κ*B p65 and ERK1/2 could be inhibited by C23 (Figure 4(b)). Additionally, we found that C23 could play the same role in an in vitro psoriasis-like environment. We created a psoriasis-like environment in keratinocytes by treating HaCaT and HEKa cells with 10 ng/ml M5 for 24 hours. Psoriatic cells were then either left untreated or pretreated with 1 *μ*g/ml C23 for 30 minutes and then stimulated with 1 *μ*g/ml rhCIRP for 1 hour. After rhCIRP stimulation, cells were collected for qRT-PCR and western blot analysis. As shown in Figure 5, pretreatment of psoriatic keratinocytes with C23 significantly reduced rhCIRP-induced TNF-*α*, IL-6, and IL-8 expressions and phosphorylation of NF-*κ*B p65 and ERK1/2 compared to that in psoriatic keratinocytes administered rhCIRP only.

### 3.4. Effects of C23 on IMQ-Induced Psoriatic Mice

**Figure 6:**
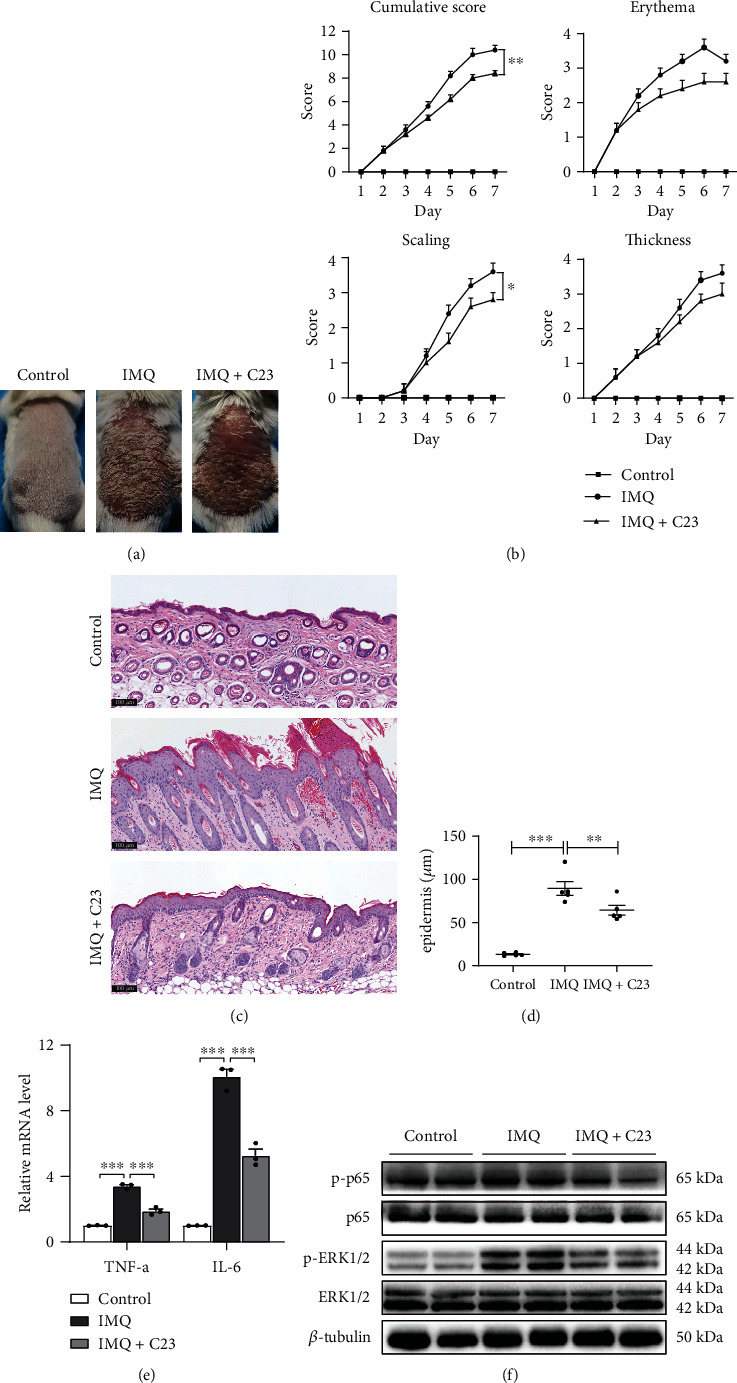
Effects of C23 on IMQ-induced psoriatic mice. (a) Representative images of each group. (b) Single (erythema, scaling, and thickness) and cumulative scores of skin lesions in each group (*n* = 5 mice/group). (c) Representative H&E staining of back skin sections of each group. Scale bar = 100 *μ*m. (d) Microscopic quantification of epidermal hyperplasia in the back skin of mice in each group (*n* = 5 mice/group). (e) qRT-PCR analysis of TNF-*α* and IL-6 mRNA levels in the back skin of mice in each group (*n* = 3). (f) Western blot analysis of the phosphorylation of NF-*κ*B p65 and ERK1/2 in skin tissues from the three groups. Data are expressed as the mean ± SEM. ^∗^*P* < 0.05, ^∗∗^*P* < 0.01, and ^∗∗∗^*P* < 0.001.

To assess the therapeutic effect of blocking eCIRP on psoriasis in vivo, we administered C23 intraperitoneally to IMQ-induced psoriatic mice. We took photographs of the skin lesions, and two dermatologists scored the mice according to the method of Moos et al. [26]. The C23 therapy reduced the cumulative score and scaling score in IMQ-treated psoriatic mice (Figures 6(a) and 6(b)). In addition, H&E staining showed that mice in the IMQ group exhibited pronounced epidermal hyperplasia, whereas epidermal thickness was reduced in mice in the C23-treated group compared to mice in the IMQ group (Figures 6(c) and 6(d)). We then examined the expression of TNF-*α* and IL-6 mRNA in the skin lesions of the mice. As shown in Figure 6(e), TNF-*α* and IL-6 expressions were significantly upregulated in IMQ-induced psoriatic mice, while the intraperitoneal injection of C23 reduced the expression of TNF-*α* and IL-6 mRNA in the skin lesions. In addition, C23 treatment reduced the phosphorylation of NF-*κ*B p65 and ERK1/2 (Figure 6(f)). These data indicate that C23 can decrease local skin inflammation by inhibiting the proinflammatory effect of eCIRP.

## 4. Discussion

In addition to its intracellular function as an RNA-binding protein to regulate gene expression [27–29], multiple studies have reported that CIRP can be secreted extracellularly and that eCIRP plays important roles in acute and chronic inflammatory diseases. A recent study showed that serum eCIRP levels were upregulated in patients with psoriasis [30], which is consistent with our results. In addition, we found that eCIRP levels were upregulated both in IMQ-induced psoriatic mice and M5-induced psoriatic keratinocytes, and the former could be inhibited by topical calcipotriol. Studies from our laboratory and other laboratories have undoubtedly shown that eCIRP levels are strongly associated with psoriasis. However, the specific role of eCIRP in the development of psoriasis and the mechanisms involved remain unclear.

Here, we found that eCIRP was able to enhance the expression of TNF-*α*, IL-6, and IL-8 in both HaCaT and HEKa cell lines. Similar regulation of the inflammatory response by eCIRP has been observed in other studies [31–33]. In models of hemorrhagic shock and sepsis, eCIRP can act as an important endogenous proinflammatory mediator that directly upregulates the expression of TNF-*α* and IL-6, triggering a waterfall response of inflammation and exacerbating tissue damage, while neutralizing antibodies of eCIRP could attenuate the inflammatory response and improve survival in hemorrhagic and septic animals [34, 35]. Taken together, the proinflammatory effect of eCIRP plays an important role in the development of psoriasis.

To further explore how eCIRP upregulates the expression of proinflammatory cytokines, we investigated the downstream signaling pathways of eCIRP. Our results confirmed that eCIRP is a key inducer of the NF-*κ*B p65 and ERK1/2 signaling pathways in keratinocytes. Certainly, understanding the signaling pathways whose activation by eCIRP in keratinocytes results in an inflammatory response will impact the search for new targets and therapeutic strategies for psoriasis. NF-*κ*B signaling pathway activation is closely associated with psoriasis development [36, 37] as well as the increased activation of ERK1/2 [25, 38] in psoriatic skin. In other inflammatory diseases, eCIRP can also exert proinflammatory effects by activating these two signaling pathways. Zhou et al. [39] suggested that eCIRP similarly activates the NF-*κ*B signaling pathway in BV2 cells. Likewise, Ran et al. [40] indicated that CIRP is expressed in the bronchi of chronic obstructive pulmonary disease (COPD) patients and is involved in proinflammatory cytokine expression after cold stimulation via the ERK and NF-*κ*B pathways. Therefore, eCIRP may be involved in regulating keratinocyte inflammatory microenvironment formation through activation of the NF-*κ*B and ERK1/2 signaling pathways.

C23, a CIRP-derived 15-mer peptide, competitively binds receptors for CIRP, such as the TLR4/MD2 complex, LPS, and HMGB1 [41]. Preincubation with C23 was found to prevent rhCIRP-induced release of TNF-*α* from the human monocyte THP-1 cell line [10], suggesting that C23 blocks CIRP activity. As in the THP-1 cell line, C23 inhibited eCIRP-induced TNF-*α*, IL-6, and IL-8 expressions in HaCaT and HEKa cell lines. Moreover, we discovered that pretreatment of the two cell lines with C23 effectively inhibited eCIRP-induced downstream activation of the NF-*κ*B p65 and ERK1/2 pathways. Then, we verified the results in a psoriasis-like model in vitro by stimulating keratinocytes with M5. We found that C23 could play the same role in an in vitro psoriasis-like environment.

Furthermore, by intraperitoneal injection of C23 into IMQ-induced psoriatic mice, we found that C23 strongly inhibited psoriatic lesions in the mice. TNF-*α* and IL-6 mRNA levels in skin lesions were downregulated in C23-treated IMQ-induced psoriatic mice. This finding is consistent with several other studies. Denning et al. [42] indicated that C23 treatment of neonatal sepsis resulted in a reduction in the proinflammatory cytokines IL-6 and IL-1*β* in serum and lung tissue, respectively. McGinn et al. [21] observed that pretreatment with C23 significantly decreased the levels of recombinant mouse CIRP- (rmCIRP-) induced TNF-*α* in a dose-dependent fashion in cultured macrophages and in C23-treated mice with renal I/R. Additionally, researchers found that the serum and intestinal tissue levels of TNF-*α* were elevated in vehicle-treated mice with intestinal I/R but decreased in C23-treated control mice [43]. Moreover, psoriasis is characterized by increased expression of TNF-*α* and IL-6 in lesions [44], and TNF-*α* is a verified drug target in psoriasis [45, 46] and other autoimmune diseases, such as inflammatory bowel disease (IBD) [47] and RA [48]. Similarly, C23 may protect against an exaggerated inflammatory response in psoriasis by reducing eCIRP-triggered proinflammatory cytokine expression via the NF-*κ*B p65 and ERK1/2 pathways. Thus, further evidence of the role of eCIRP in psoriasis is provided. The use of eCIRP as a target, as with C23, to inhibit its binding to the corresponding receptor may represent a novel treatment for psoriasis.

However, the formation of an inflammatory microenvironment is important for the development of psoriasis, and keratinocyte hyperproliferation is also a major pathological feature of psoriasis. We will further investigate this notion in a follow-up study and observe the effects of eCIRP and C23 on keratinocyte proliferation.

## 5. Conclusions

In conclusion, eCIRP was elevated in patients with psoriasis, as well as in IMQ-induced psoriatic mice and M5-stimulated psoriatic cells. eCIRP upregulated the expression of proinflammatory cytokines, which could be suppressed by C23. Taken together, this work demonstrated that eCIRP may be involved in the pathogenesis of psoriasis and therefore may serve as a new therapeutic strategy in psoriatic treatment. Further studies will consider the role and mechanism of eCIRP in keratinocyte proliferation.

## Data Availability

The datasets used and/or analyzed during the current study are available from the corresponding author on reasonable request.

## Supplementary Materials

Supplementary Materials See the Supplementary Table in the Supplementary Material for the sequences of primers used for qRT-PCR in this study.
