# Supplementary material for: Extracellular CIRP Upregulates Proinflammatory Cytokine Expression via the NF-kappaB and ERK1/2 Signaling Pathways in Psoriatic Keratinocytes
Source: Mediators Inflamm. 2022 Sep 6;2022:5978271. doi: 10.1155/2022/5978271 (PMC9470347; doi:10.1155/2022/5978271)
Supplement: Supplementary Materials — See the Supplementary Table in the Supplementary Material for the sequences of primers used for qRT-PCR in this study. [file 5978271.f1.docx]

**Supplementary Table**

Table S1: The sequences of primers for qRT-PCR used in this study

| Name | Sequences |
| --- | --- |
| h-TNF-α | Foward: 5`-TCCTTCAGACACCCTCAACC-3` |
|  | Reverse: 5`-AGGCCCCAGTTTGAATTCTT-3` |
| h-IL-6 | Foward: 5`-AAGCCAGAGCTGTGCAGATGAGTA-3` |
|  | Reverse: 5`-TGTCCTGCAGCCACTGGTTC-3` |
| h-IL-8 | Foward: 5`-GTCCTTGTTCCACTGTGCCT-3` |
|  | Reverse: 5`-GCTTCCACATGTCCTCACAA-3` |
| h-GAPDH | Foward: 5`-AGGTCCACCACTGACACGTT -3` |
|  | Reverse: 5`-GCCTCAAGATCATCAGCAAT -3` |
| m-TNF-α | Foward: 5`-ATGTCTCAGCCTCTTCTCATTC-3` |
|  | Reverse: 5`-GCTTGTCACTCGAATTTTGAGA-3` |
| m-IL-6 | Foward: 5`- CTCCCAACAGACCTGTCTATAC -3` |
|  | Reverse: 5`-CCATTGCACAACTCTTTTCTCA -3` |
| m-β-actin | Foward: 5`-CTACCTCATGAAGATCCTGACC-3` |
|  | Reverse: 5`-CACAGCTTCTCTTTGATGTCAC-3` |
